# Rolling With Host Immunity: Virulence Beyond The Glycolysis

**DOI:** 10.3389/fcimb.2022.939828

**Published:** 2022-06-15

**Authors:** Sen Guo, Dong Sun

**Affiliations:** General Surgery, Qilu Hospital of Shandong University, Jinan, China

**Keywords:** *Coxiella burnetii*, type IV secretion system, NF-κB, phosphatase, sugar metabolism

In contrast to functional redundancy of the *Legionella pneumophila* type IV secretion system (T4SS) effectors (Song et al., 2021; Fu et al., 2022a; Song et al., 2022), deletion of a single T4SS effector coding gene results in substantially less virulence of *Coxiella burnetii* (Weber et al., 2013; Fu et al., 2022b), the causative agent of Q fever. Elucidating the biological functions of T4SS effectors of *C. burnetii* lays a prerequisite for the development of anti-infection drugs, which is of great significance for treating and preventing Q fever. However, little is known about the biochemical activities of *C. burnetii* effector proteins.

NF-κB regulates several signaling pathways critical to immunity, cell proliferation and apoptosis, making them common targets for pathogens that manipulate host cell function (Rahman and McFadden, 2011). NF-κB has been demonstrated to be inhibited in the late stage of *C. burnetii* infection (Mahapatra et al., 2016). This inhibition has been attributed to virulence proteins transported by the bacterial Dot/Icm system (Mahapatra et al., 2016) but the specific mechanism remains unclear. In order to address this issue, Zhang et al. (2022) screened the regulatory activities of *C. burnetii* effectors library in NF-κB signalling pathway using the NF-κB reporter system, and discovered that the effector protein CinF (Cbu_0513) inhibits NF-κB activation effectively. Surprisingly, by the bioinformatic analysis, CinF showed high similarities to ST0318, a fructose-1,6-bisphosphate (FBP) aldolase/phosphatase found in numerous bacteria, especially the certain Archaea species (Fushinobu et al., 2011). However, CinF lacks phosphatase activity against FBP despite being highly similar to ST0318 and is unable to dephosphorylate when p-nitrophenyl phosphate is used as the substrate. The biological functions of CinF and ST0318 are therefore different, even though their structures are similar. Further experiments have demonstrated that CinF is a protein phosphatase that exhibits high specificity towards IκBα.

In conclusion, the results of this study confirmed that CinF is a novel protein phosphatase, and the target of CinF in host cells is the key regulatory protein IκBα in the NF-κB immune pathway. Dephosphorylation of IκBα prevents itself from being degraded by proteases, thus inhibiting NF-κB activation during infection, which is critical to the survival and proliferation of the pathogen. This study not only identified a *C. burnetii* effector protein that inhibits NF-κB, filling a gap in previous studies, but also systematically discussed the biological significance of this protein in bacterial infection and intracellular growth. The study provides a solid foundation for studying the infection mechanism of intracellular pathogens.

Although CinF is structurally similar to ST0318, their biochemical activities are quite different. Protein is the substrate of this new phosphatase rather than carbohydrates or lipids. This study also suggests that metabolic enzymes such as those involved in glycolysis of distantly related microorganisms have been acquired by *C. burnetii* through horizontal gene transfer, and evolved into proteins that are capable of interfering with the immune response, expanding the current knowledge of mechanisms utilized by pathogens to evade host immunity.

## Author Contributions

SG, Conceptualization, Writing – original draft; DS, Conceptualization, Writing – review and editing. All authors contributed to the article and approved the submitted version

## Conflict of Interest

The authors declare that the research was conducted in the absence of any commercial or financial relationships that could be construed as a potential conflict of interest.

## Publisher’s Note

All claims expressed in this article are solely those of the authors and do not necessarily represent those of their affiliated organizations, or those of the publisher, the editors and the reviewers. Any product that may be evaluated in this article, or claim that may be made by its manufacturer, is not guaranteed or endorsed by the publisher.
